# Association analysis between Acetyl-Coenzyme A Acyltransferase-1 gene polymorphism and growth traits in Xiangsu pigs

**DOI:** 10.3389/fgene.2024.1346903

**Published:** 2024-05-02

**Authors:** Meimei Xiao, Yong Ruan, Jiajin Huang, Lingang Dai, Jiali Xu, Houqiang Xu

**Affiliations:** ^1^ Key Laboratory of Animal Genetics, Breeding and Reproduction in the Plateau Mountainous Region, Ministry of Education, Guizhou University, Guiyang, China; ^2^ Guizhou Provincial Key Laboratory of Animal Genetics, Breeding and Reproduction, Guizhou University, Guiyang, China; ^3^ College of Animal Science, Guizhou University, Guiyang, China

**Keywords:** Xiangsu pigs, *ACAA1*, single-nucleotide polymorphism, fat deposition, growth traits

## Abstract

**Introduction:**

Acetyl-Coenzyme A Acyltransferase-1 (*ACAA1*) is a peroxisomal acyltransferase involved in fatty acid metabolism. Current evidence does not precisely reveal the effect of the *ACAA1* gene on pig growth performance.

**Methods:**

The present study assessed the mRNA expression levels of the *ACAA1* gene in the heart, liver, spleen, lung, kidney of 6-month-old Xiangsu pigs and in the longissimus dorsi muscle at different growth stages (newborn, 6 months and 12 months of age) using RT-qPCR. The relationship between single-nucleotide polymorphisms (SNPs) of *ACAA1* gene and growth traits in 6-month-old and 12-month-old Xiangsu pigs was investigated on 184 healthy Xiangsu pigs using Sanger sequencing.

**Results:**

The *ACAA1* gene was expressed in heart, liver, spleen, lung, kidney, and longissimus dorsi muscle of 6-month-old pigs, with the highest level of expression in the liver. *ACAA1* gene expression in the longissimus dorsi muscle decreased with age (*p* < 0.01). In addition, four SNPs were identified in the *ACAA1* gene, including exon g.48810 A>G (rs343060194), intron g.51546 T>C (rs319197012), exon g.55035 T>C (rs333279910), and exon g.55088 C>T (rs322138947). Hardy-Weinberg equilibrium (*p* > 0.05) was found for the four SNPs, and linkage disequilibrium (LD) analysis revealed a strong LD between g.55035 T>C (rs333279910) and g.55088 C>T (rs322138947) (*r* ^*2*^ = 1.000). Association analysis showed that g.48810 A>G (rs343060194), g.51546 T>C (rs319197012), g.55035 T>C (rs333279910), and g.55088 C>T (rs322138947) varied in body weight, body length, body height, abdominal circumference, leg and hip circumference and living backfat thickness between 6-month-old and 12-month-old Xiangsu pigs.

**Conclusion:**

These findings strongly demonstrate that the *ACAA1* gene can be exploited for marker-assisted selection to improve growth-related phenotypes in Xiangsu pigs and present new candidate genes for molecular pig breeding.

## 1 Introduction

Pork is one of the important sources of animal protein for humans. Improving the growth traits of pigs is an ongoing goal in the field of animal husbandry. Growth traits such as living backfat thickness (LBT), body length (BL), body height (BH), chest circumference (CC), chest depth (CD), and rump circumference (RC) are directly related to the economic efficiency of pigs (Liu et al., 2021; Zhang et al., 2021).Growth traits are quantitative traits that are regulated by a few major genes and a large number of minor genes (Boyle et al., 2017). With the rapid development of molecular breeding and sequencing technologies, many genes that regulate pig growth traits have been identified and confirmed (Shi et al., 2022).

Acetyl-Coenzyme A Acyltransferase-1 (*ACAA1*) cleaves 3-ketoacyl-CoA to acetyl-CoA and acyl-CoA by catalyzing the β-oxidation of fatty acids in peroxisomes, driving the synthesis and secretion of fatty acids (Wanders et al., 2001; Wang et al., 2021). This enzyme is also key in regulating fatty acid oxidation and lipid metabolism (Luo et al., 2018). The *ACAA1* gene is downstream in the peroxisome proliferator–activated receptor (PPAR) signaling pathway. The *PPAR* enzyme critically regulates fatty acid synthesis and transport, catalyzes the synthesis of esterified cholesterol from free cholesterol and long-chain fatty acids, and plays a crucial role in fatty acid metabolism (Li et al., 2017). Recent research on the *ACAA1* gene has primarily focused on human cancer and metabolic diseases. Emerging evidence indicates that *ACAA1* gene expression is downregulated in hepatocellular carcinoma and renal clear cell carcinoma (Li et al., 2017; Liu et al., 2015; Nwosu et al., 2018; Yan et al., 2017; Zhang et al., 2019). The *ACAA1* gene was revealed to be highly expressed in triple-negative breast cancer cells, and inhibiting the *ACAA1* gene decreased the proliferation of triple-negative breast cancer cells (Peng et al., 2023). *ACAA1* is a type 2 diabetes (T2D) biomarker that can predict the metabolic characteristics of pre-diabetes in mouse models (Kumar et al., 2015). Research on the *ACAA1* gene in animal husbandry has linked ACAA1 mutation to milk production traits of buffalo. Analysis of the liver transcriptomes and the microarray dataset of Hereford (beef breed) and Holstein-Friesian (dairy breed) bulls with different genetic backgrounds revealed that Hereford bulls were highly involved in fatty acid biosynthesis and lipid metabolism by up-regulating *ACAA1* gene expression as compared to Holstein-Friesian (Lisowski et al., 2014).

Single Nucleotide Polymorphism (SNP) refers to the DNA sequence polymorphism caused by single nucleotide variation at the chromosome genomic level, and the frequency of this variation is more than 1% in at least one population (Taylor et al., 2001; Vignal et al., 2002). Five SNPs (g.-681 A>T, g.-24348 G>T, g.-806 C>T, g.-1868 C>T and g.-23117 C>T) were identified in the buffalo *ACAA1* gene, among which g.-681 A>T, g.-24348 G>T, and g.-23117 C>T are significantly associated with milk production traits in buffaloes. In addition, the g.-681 A>T mutation in the promoter region significantly changed the transcriptional activity (Deng et al., 2023). A missense variant rs117916664 of the *ACAA1* gene was identified in a Han Chinese early-onset familial Alzheimer’s disease (AD) family and found to be associated with early-onset familial AD (Luo et al., 2021). A genetic polymorphism in the *ACAA1* gene alters the association between endotoxin exposure and asthma (Sordillo et al., 2011). However, data on the polymorphism of the *ACAA1* gene in pigs is scarce.

Xiangsu pig is a novel breeding strain that utilizes Sutai pig and Congjiang Xiang pig as parents and repeatedly backcrossed with Congjiang Xiang pig as male parent. Congjiang Xiang pig has early sexual maturity, strong fat deposition capacity, and strong disease resistance but slow growth (Liu et al., 2018; Tang et al., 2018; Xu et al., 2022). The SuTai pig is a breed characterized by its high reproductive rate and strong adaptability (Bao et al., 2012). The Congjiang xiang pig accounts for 87.5% of the genetic lineage within the Xiangsu pig population, allowing for the full inheritance of its genetic traits in subsequent generations (Xu et al., 2022). Therefore, we selected *ACAA1* gene as a candidate gene for the growth traits of the Xiangsu pig and evaluated the relationship between *ACAA1* gene polymorphism and the growth traits of the Xiangsu pig, which is valuable for Xiangsu pig breeding in the future.

## 2 Materials and methods

### 2.1 Experimental animals

The animal experiments fully adhered to the guidelines of the Animal Welfare Committee of Guizhou University (EAE-GZU-2022-E031). The production cycle (farrowing to growing-finishing) of Xiangsu pig is 12 months. A total of 184 healthy Xiangsu pigs under the same feeding level were selected to track and record the growth traits (body weight, body length, body height, chest circumference, abdominal circumference, tube circumference, leg and hip circumference and living backfat thickness) of 6-month-old and 12-month-old Xiangsu pigs. The measurement method of body weight, body length, body height, chest circumference, abdominal circumference, tube circumference, leg and hip circumference was referred to as NY/T2894-2016. The probe of the handheld veterinary ultrasound diagnostic device (KX5200) had been positioned vertically on the 10th and 11th thoracic vertebrae of pigs to measure the living backfat thickness of 6-month-old and 12-month-old pigs.

### 2.2 Primer design

The upstream and downstream primers were designed by Primer Premier 5.0 software using the pig *ACAA1* gene (accession number: NC_010455.5) and mRNA sequence (accession number: XM_003132103.4); *GADPH* gene (accession number: NC_010447.5) and mRNA sequence (accession number: NM_001206359.1) available in NCBI GeneBank. The primers were synthesized by Beijing Qingke Biotechnology Co., Ltd., (Table 1).

**TABLE 1:** Primer information of *ACAA1* gene sequence.

Primer names	Primer sequences (5′→3′)	Product size/bp	Annealing temperature/°C
ACAA1-Exon7	F:GACTCTTCAGAGGAAGAGAGAGGAG	649	57
	R:CAGCAGACGATGACTCTGCTGAT		
ACAA1-Exon9	F:TTGTTAGATGTGTCCTTCACTGTGG	675	63
	R:TCAACTTTCTAGGCCTCCAGAGTT		
ACAA1-Exon15	F:TGAGGTCTGGCATCTTCTGTGC	615	63
	R:CTCAGAGGTGGAGCAGTACAAAGAG		
ACAA1-qPCR	F:ATGGGGATAACCTCAGAGAACGT	175	55
	R:TCTCATTGCCCTTGTCATCGTAG		
GADPH	F:GGTCGGAGTGAACGGATTT	247	60
	R:CCATTTGATGTTGGCGGGA		

Note: F denotes the upstream primer, and R denotes the downstream primer. bp: base pair.

### 2.3 Collection of blood and tissue samples

The blood (5 mL) of 184 3-month-old Xiangsu pigs was drawn through the jugular vein using an EDTA anticoagulant tube, labeled with the number and date, and stored in a refrigerator at −20°C for DNA extraction. Three 6-month-old Xiangsu pigs were randomly selected from Xiangsu pigs for slaughter. The heart, liver, spleen, lung, kidney and longissimus dorsi muscle were collected in 2 mL cryopreservation tubes and stored in a refrigerator at −80°C for RNA extraction in order to compare the expression of *ACAA1* gene in different tissues of 6-month-old pigs. Three pigs are selected for slaughter from each age group (newborn and 12 months old) at each stage, and the longissimus dorsi muscle was collected and preserved in 2 mL cryopreservation tubes in a refrigerator at −80°C for RNA extraction in order to compare the expression of *ACAA1* at different ages.

### 2.4 DNA and RNA extraction

DNA was extracted from 184 blood samples of Xiangsu pigs using the whole blood DNA extraction kit (D3392-01, Omega). Total RNA of heart, liver, spleen, lung and kidney of Xiangsu pigs at 6 months of age and total RNA of longissimus dorsi muscle at newborn, 6 months and 12 months of age was extracted using the TRIzol Extraction Kit (15,596,026; Thermo Fisher). The concentration and purity of DNA and RNA were determined using an ultra-micro spectrophotometer (Thermo Mano Drop 2000), followed DNA by storage at −20°C and RNA by storage at −80°C (Supplementary Table S1).

### 2.5 cDNA synthesis

cDNA was synthesized using the RNA Reverse Transcription Kit (A234-10; GenStar). By this kit 1 μg RNA, 1 μL Primer Mix, 10 μL 2× StarScript III Buffer, 1 μL StarScript III Enzyme Mix, and then supplemented with Nuclease-free Water to a final volume of 20 μL. The mixture was incubated at 50°C for 15 min, followed at 85°C for 5 min. The resulting cDNA was stored at −20°C for subsequent experiments.

### 2.6 Amplification

A 30 μL system was used for PCR amplification. The PCR reaction mixture was prepared as follows: 15 μL 2×Taq PCR Starmix, 10.5 μL ddH_2_O, 1.5 μL DNA template (40 ng/μL), forward and reverse primers, 1.5 μL each. The PCR amplification procedure included a pre-denaturation at 94°C for 3 min; after 35 cycles, 94°C denaturation for 30 s (Tm see Table 1), annealing for 30 s, 72°C extension for 1 min; 72°C final extension for 5 min; infinite hold at 4°C. The PCR products (184) were visualized with 1% agarose gel electrophoresis and sent to Qingke Biological Co., Ltd. for sequencing.

### 2.7 Real-time fluorescent quantitative PCR

The total RNA concentration was standardized (1000 ng/μL), and 1 μg total RNA, 2 μL 5 × gDNA Eraser Buffer gDNA Eraser, and RNase-Free water were added to the enzyme-free PCR tube to obtain a 10 μL reaction volume. The samples were incubated at 37°C for 5 min to remove gDNA. In addition, a 10 μL Master Mix (including 1 μL PrimeScript RT Enzyme Mix I, 1 μL RT Primer Mix, 4 μL 5× PrimeScript Buffer 2, and 4 μL RNase-Free ddH_2_O) was prepared on ice. Total RNA (without gDNA) and Master Mix were mixed in an enzyme-free PCR tube, incubated at 42°C for 15 min, then at 85°C for 5 min, and the resultant cDNA was stored at −20°C.

A 10 μL real-time fluorescence quantitative PCR reaction mixture was constituted as follows: 5 μL 2× PowerUp SYBP Green Master Mix (A25742; Thermo Fisher), 0.5 μL cDNA template, 0.4 μL (10 μmol/L) forward and reverse primers, and 3.7 μL ddH_2_O. The quantitative real-time PCR amplification steps were set as follows: 50°C UDG enzyme activation 2 min; pre-denaturation at 95°C for 2 min; 95°C denaturation 15 s, 55°C annealing 30 s, 72°C extension 30 s, 40 cycles; from 72°C to 95°C, a temperature increase step by 1.6°C per second for 15 s, and then a temperature decrease step by 1.6°C per second from 95°C to 60°C. Each sample had 3 replicates.

### 2.8 Statistical analysis

The presence of SNPs in *ACAA1* sequence was determined via peak plotting against the PCR sequencing reads using the SeqMan software. The genotype, allele frequency, and Hardy-Weinberg equilibrium (HWE) of each mutation site were computed directly, and whether the genotype conformed to HWE was analyzed using the χ2 test and *p*-value. Nei’s method was employed to analyze the genetic indexes of the population, including gene heterozygosity (*He*), gene homozygosity (*Ho*), and polymorphism information content (*PIC*) (Nei and Roychoudhury, 1974). The effective number of alleles (*Ae*) is related to the distribution of gene frequency in the population, and the markers were calculated with GenAlEx 6.5 (New Brunswick, NJ, United States) (Peakall and Smouse, 2012). *PIC* refers to a measure used to assess the ability to detect polymorphism among individuals in a population. The range of polymorphic information content is between 0 and 1, with higher values indicating greater information content and polymorphism in genetic markers (Serrote et al., 2020). The SHEsis platform (http://analysis.bio-x.cn) was used for linkage disequilibrium (LD) analysis and haplotype analysis of single-nucleotide polymorphisms (SNPs) in the *ACAA1* gene (Li et al., 2009; Shi and He, 2005). Squared allele-frequency correlations (*r* ^*2*^) and Standardized disequilibrium coefficients (*D′*) were used to estimate the level of LD (Du et al., 2007). The *r* ^*2*^ value is commonly used to evaluate the degree of linkage disequilibrium. When *r* ^*2*^ > 0.33, it is a strong linkage disequilibrium state (Ardlie et al., 2002). *D′* is the normalized coefficient of linkage disequilibrium (LD) divided by the theoretical maximum difference between the observed and expected allele frequencies (Bozorgmehr et al., 2020). Haplotype and diplotypes analyses were performed based on SNPs.

*ACAA1* genotype association analysis was performed using IBM SPSS 22.0 (IBM, New York, NY, United States). The least square method was applied to the general linear model (GLM) to examine the association between genotypes and growth traits of 184 Xiangsu pigs. A statistical model, *Y*_*ij*_ *= μ+G*_*i*_ *+ Sj + e*_*ij*_, was developed where *Y*_*ij*_ denotes the observed growth trait; *μ* denotes the overall population means; *G*_*i*_ represents the fixed effect of the genotype, *Sj* represents the random effect of sire, and *eij* denotes the random error (Naicy et al., 2017).

The relative expression of the *ACAA1* gene was calculated using the 2^−ΔΔCt^ method (Saitou and Nei, 1987), with glyceraldehyde-3-phosphate dehydrogenase (*GAPDH*) gene as the endogenous reference gene, where ∆ Ct = Ct (target gene)-Ct (*GADPH*), and 2^−ΔΔCt^ represents the differetial expression multiple relative to *GADPH* expression A one-way analysis of variance (ANOVA) was conducted to compare the differences among the heart, liver, spleen, lung, kidney, and longissimus dorsi muscle of 6-month-old pigs. Additionally, ANOVA was performed to assess the differences in the longissimus dorsi muscle of newborns, 6-month-old, and 12-month-old pigs.

## 3 Results

### 3.1 Expression level of *ACAA1* gene in Xiangsu pig tissues

**FIGURE 1:**
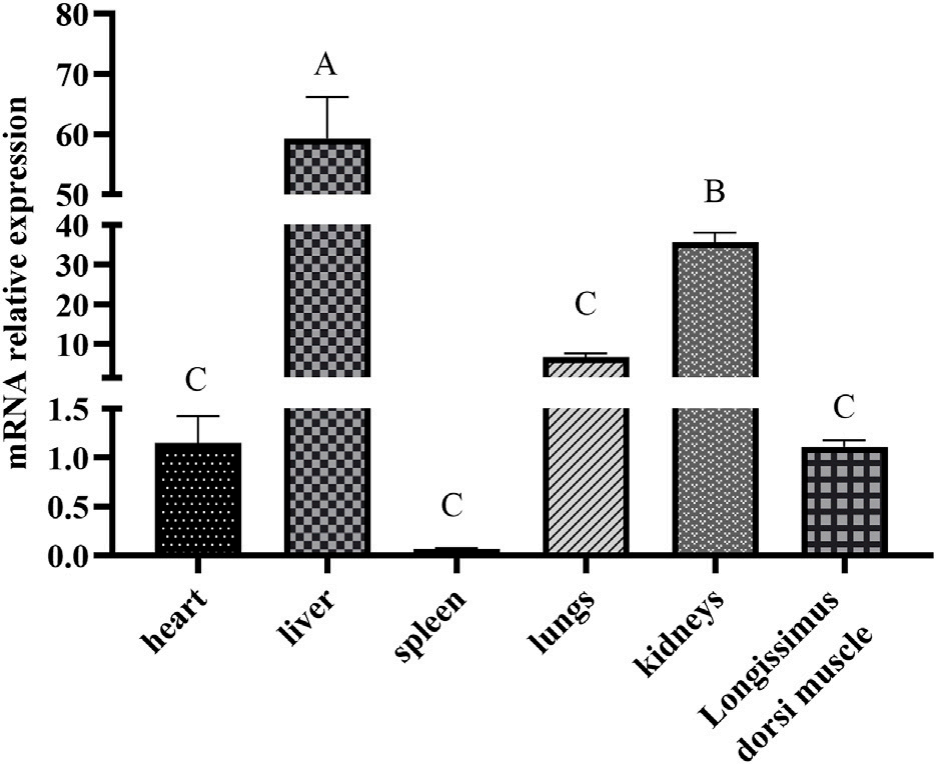
Analysis of the differential expression of the *ACAA1* gene in various tissues at 6 months of age. Note: Relative mRNA expression levels were calculated by 2^−ΔΔCt^ method. “A, B, C” indicate extremely significant differences among different tissues of six-month-old Xiangsu pigs (*p* < 0.01); the same uppercase letters indicate no significant difference.

**FIGURE 2:**
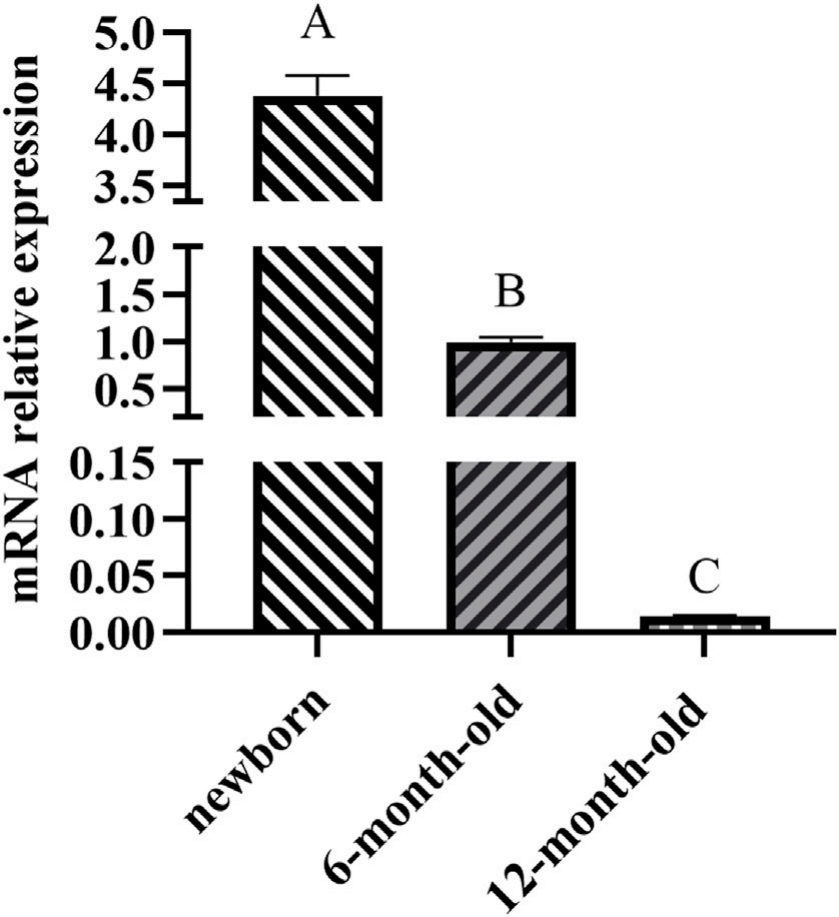
Analysis of the differential expression of the *ACAA1* gene in dorsal longest muscle at newborn, 6 months, and 12 months of age. Note: Relative mRNA expression levels were calculated by 2^−ΔΔCt^ method. “A, B, C” denotes extremely significant differences in dorsal longest muscle at birth, 6 months and 12 months of age (*p* < 0.01).

The *ACAA1* gene was expressed in the heart, liver, spleen, lung, kidney, and longissimus dorsi muscle of 6-month-old Xiangsu pigs, with higher levels in the liver and kidney and lower levels in the spleen and longissimus dorsi muscle (*p* < 0.01) (Figure 1). Newborn piglets exhibited the highest expression of the *ACAA1* gene (*p* < 0.01) in the longissimus dorsi muscle, and it decreased with age (Figure 2).

### 3.2 Analysis of the *ACAA1* gene polymorphism

**FIGURE 3:**
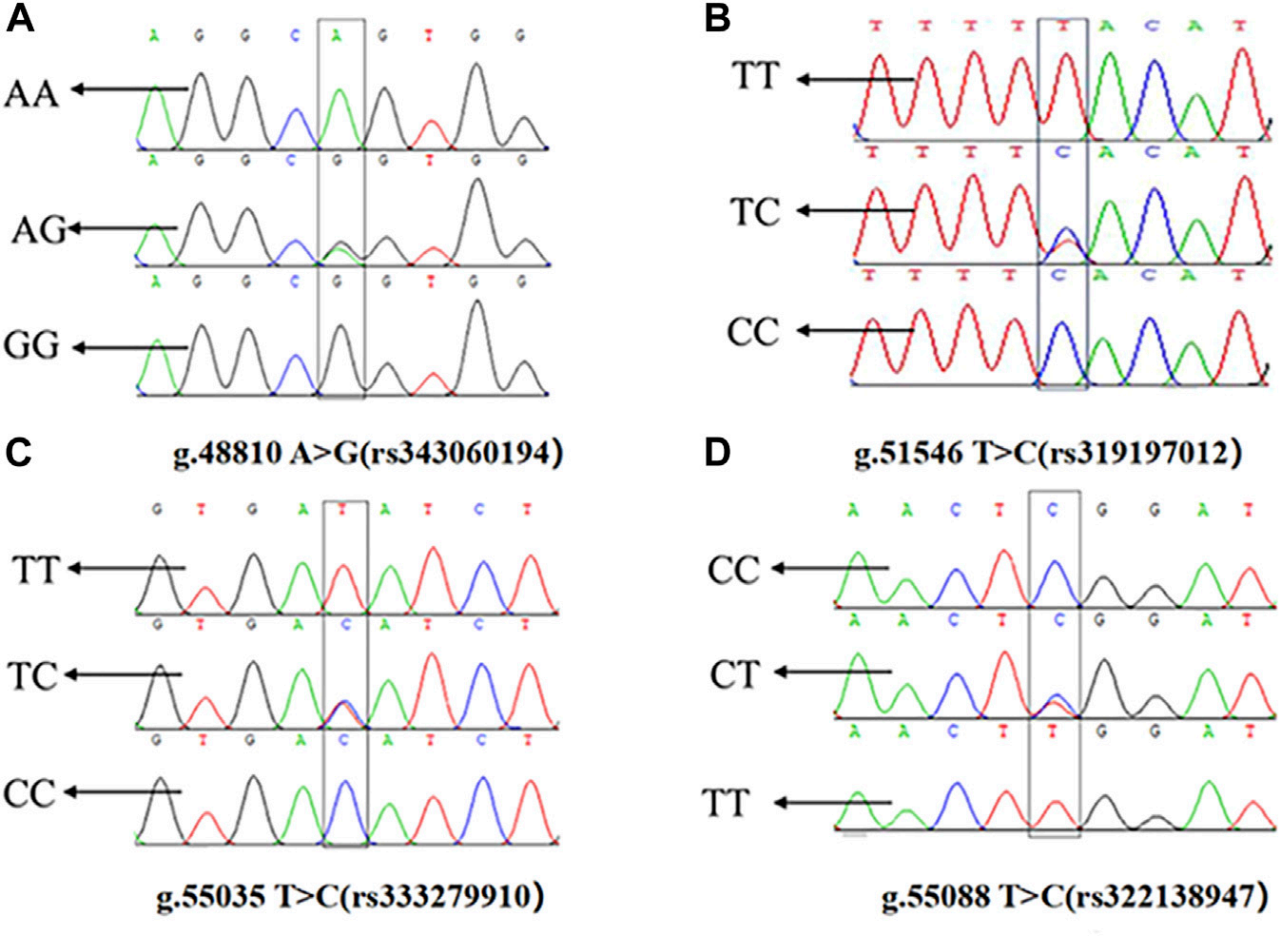
Sequencing peaks of four SNPs in the *ACAA1* gene. **(A)** g.48810 A>G (rs343060194), **(B)** g.51546 T>C (rs319197012), **(C)** g.55035 T>C (rs333279910), **(D)** g.55088 C>T (rs322138947).

The presence of SNPs in *ACAA1* gene sequence was determined via peak plotting against the PCR sequencing reads using the SeqMan. Four SNPs were detected in the *ACAA1* gene of Xiangsu pigs, including exon 7 g.48810 A>G (rs343060194), intron 9 g.51546 T>C (rs319197012), exon 15 g.55035 T>C (rs333279910), and exon 15 g.55088 C>T (rs322138947) (Figure 3). Using DNA Star software to compare the sequences of three exon mutation sites g.48810 A>G (rs343060194), g.55035 T>C (rs333279910), g.55088 C>T (rs322138947) with the NCBI amino acid reference sequence of the ACAA1 (XP_003132151.1). Three exon mutation sites indicated no changes in the amino acid sequence, therefore they are synonymous mutations.

### 3.3 Genetic polymorphism analysis of the *ACAA1* gene

Each mutation site had three genotypes. The chi-square test (χ2) revealed that the four SNPs loci g.48810 A>G (rs343060194), g.51546 T>C (rs319197012), g.55035 T>C (rs333279910) and g.55088 C>T (rs322138947) were in HWE (*p* > 0.05) (Table 2).

**TABLE 2:** Genotype frequencies and allele frequencies of *ACAA1* gene in Xiangsu pigs.

SNPs	rs number	Genotypic frequency			Allele frequency		χ2	*p*
g.48810 A>G	rs343060194	AA	AG	GG	A	G	3.32	0.19
		0.14 (25)	0.38 (70)	0.48 (89)	0.33	0.67		
g.51546 T>C	rs319197012	TT	TC	CC	T	C	1.87	0.39
		0.39 (72)	0.43 (79)	0.18 (33)	0.61	0.39		
g.55035 T>C	rs333279910	TT	TC	CC	T	C	1.20	0.55
		0.36 (67)	0.45 (82)	0.19 (35)	0.59	0.41		
g.55088 C>T	rs322138947	CC	CT	TT	C	T	1.20	0.55
		0.36 (67)	0.45 (82)	0.19 (35)	0.59	0.41		

Note: *p* > 0.05 indicates that the gene frequency in the population is at Hardy-Weinberg equilibrium. The number of samples is indicated in brackets.

The homozygosity (*Ho*) of the four SNPs of the *ACAA1* gene was 0.5151–0.5605, and the heterozygosity (*He*) was 0.4395–0.4849 (Table 3). The homozygosity (*Ho*) was higher than the heterozygosity (*He*), demonstrating that the four loci in this population showed a low degree of variation; effective number of alleles (*Ae*) was 1.7841–1.9413. The polymorphism information content ranged from 0.3429 to 0.3673, indicating a moderate polymorphism level (0.25<*PIC* < 0.5).

**TABLE 3:** Genetic information of *ACAA1* gene population.

SNPs	rs number	Effective allele number (*Ae*)	Homozygosity (*Ho)*	Heterozygosity (*He*)	Polymorphism information content (*PIC*)
g.48810 A>G	rs343060194	1.7841	0.5605	0.4395	0.3429
g.51546 T>C	rs319197012	1.8918	0.5225	0.4775	0.3635
g.55035 T>C	rs333279910	1.9413	0.5151	0.4849	0.3673
g.55088 C>T	rs322138947	1.9413	0.5151	0.4849	0.3673

Note: *PIC* < 0.25 is low polymorphism, 0.25<*PIC* < 0.5 is medium polymorphism, and *PIC* > 0.5 is high polymorphism.

### 3.4 Linkage disequilibrium and haplotype analysis of SNPs in the *ACAA1* gene

**FIGURE 4:**
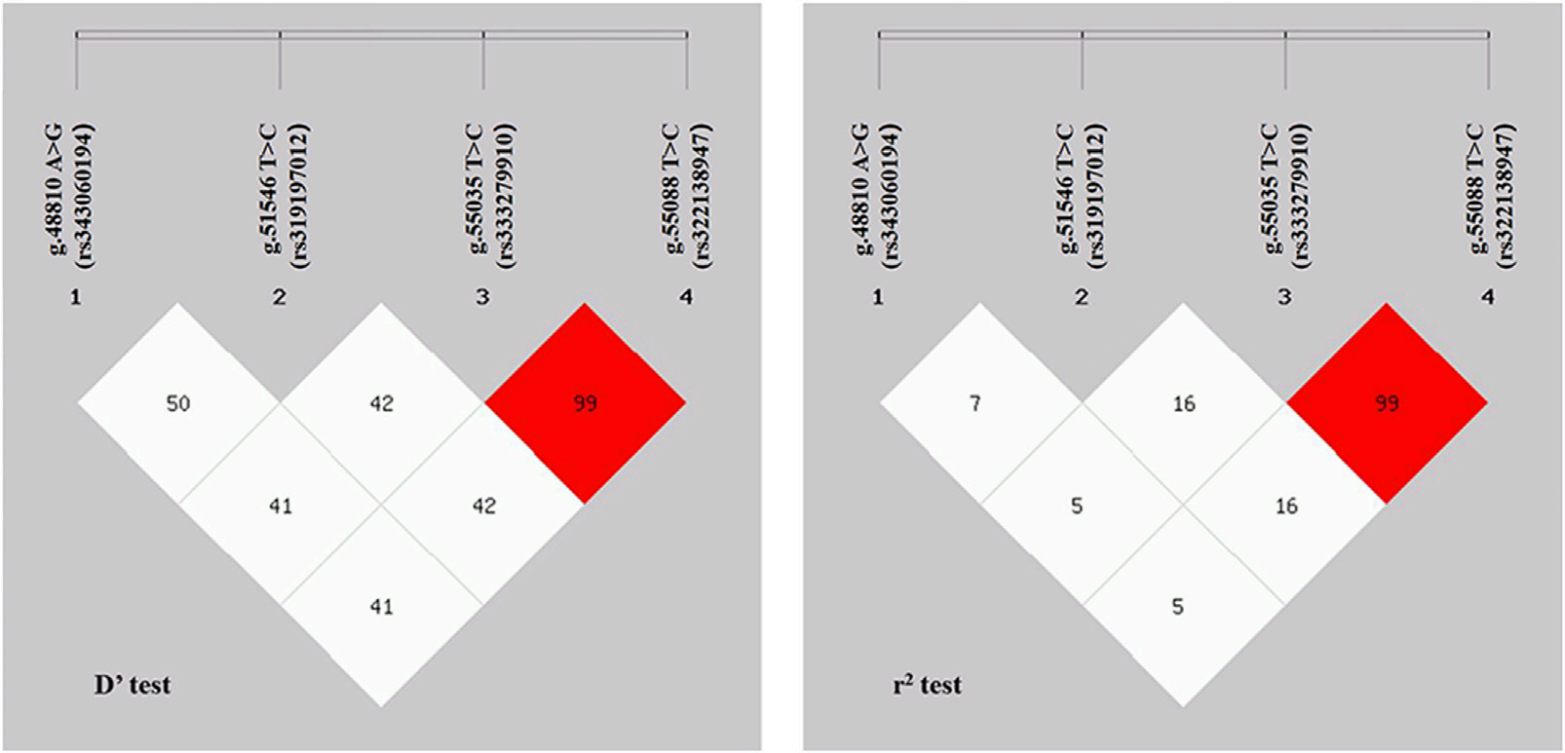
*r* ^*2*^ and *D’* values in linkage disequilibrium analysis of *ACAA1* gene SNPs.

Linkage disequilibrium (LD) analysis was performed on the four SNPs in the *ACAA1* gene (Figure 4). The *D’* values of the four SNPs ranged between 0.414 and 1.000, and the *r* ^2^ values ranged between 0.058 and 1.000. The *r* ^2^ for g.55035 T>C (rs333279910) and g.55088 C>T (rs322138947) was 1.000, indicating a strong LD.

Table 4 displays the findings of the *ACAA1* gene haplotype analysis. The population had six haplotypes with a frequency greater than 5.00%, and less than 5.00% were excluded from statistical analysis. Among the six haplotypes, Hap 1 (-GCCT-) had the highest frequency (25.00%), while Hap 6 (-ATCT-) had the lowest frequency (5.70%). Based on the paired combinations of six haplotypes, five diplotypes combinations with frequencies greater than 5.00% were obtained, including Hap1/3, -GCCT/ATTC-; Hap2/2, -GTTC/GTTC-; Hap2/5, -GTTC/GCTC-; Hap2/4, -GTTC/GTCT-; Hap1/1, -GCCT/GCCT- (Table 5).

**TABLE 4:** Haplotype frequencies of 4 SNPs in Xiangsu pig.

Haplotype	g.48810 A>G (rs343060194)	g.51546 T>C (rs319197012)	g.55035 T>C (rs333279910)	g.55088 C>T (rs322138947)	Frequency (%)
Hap1	G	C	C	T	25.00
Hap2	G	T	T	C	24.20
Hap3	A	T	T	C	21.30
Hap4	G	T	C	T	9.40
Hap5	G	C	T	C	8.80
Hap6	A	T	C	T	5.70

Note: Haplotypes with frequencies <5.00% were excluded from the analysis.

**TABLE 5:** Diplotypes and frequency of *ACAA1* gene.

Diplotypes	g.48810 A>G (rs343060194)	g.51546 T>C (rs319197012)	g.55035 T>C (rs333279910)	g.55088 C>T (rs322138947)	Frequency (%)
Hap1/3	GA	CT	CT	TC	19.02
Hap2/2	GG	TT	TT	CC	11.96
Hap2/5	GG	TC	TT	CC	7.61
Hap2/4	GG	TT	TC	CT	5.98
Hap1/1	GG	CC	CC	TT	5.43

Note: Diplotypes with frequencies <5.00% were excluded from the analysis.

### 3.5 Association analysis between *ACAA1* gene and growth performance

The association between 4 SNP loci g.48810 A>G (rs343060194), g.51546 T>C (rs319197012), g.55035 T>C (rs333279910) and g.55088 C>T (rs322138947)) and 8 growth traits at 6 months (Table 6) was examined using the SPSS 22 software. The results showed that in the 6-month-old pigs, there was a significant difference in body weight between the AG genotype and the GG genotype at the exon g.48810 A>G (rs343060194) locus (*p* < 0.05). The leg and hip circumference of the CC genotype at the intron g.51546 T>C (rs319197012) locus were significantly different from that of the TC genotype (*p* < 0.05). Furthermore, the body weight of the TC genotype at the exon g.55035 T>C (rs333279910) locus was significantly different from that of the TT genotype (*p* < 0.05), while the living backfat thickness of the TT genotype was significantly different from that of the CC genotype (*p* < 0.01). In addition, at the g.55088 C>T (rs322138947) locus, the body weight of the CT genotype was significantly different from that of the CC genotype (*p* < 0.05), and the living backfat thickness of the CC genotype was significantly different from that of the TT genotype (*p* < 0.01).

**TABLE 6:** Association analysis between *ACAA1* gene and growth traits of 6-month-old Xiangsu pigs.

SNPS	Genotypes	B W (kg)	B L (cm)	B H (cm)	C C (cm)	A C (cm)	T C (cm)	L H C (cm)	L B T (mm)
g.48810 A>G (rs343060194)	AA	70.56 ± 1.76^ab^	95.56 ± 3.11	63.76 ± 3.02	95.72 ± 3.73	96.88 ± 3.28	17.96 ± 0.79	62.88 ± 2.01	10.88 ± 0.89
	AG	70.97 ± 1.83^a^	94.97 ± 4.49	64.84 ± 4.63	95.47 ± 2.86	97.17 ± 2.86	18.00 ± 0.92	62.20 ± 2.04	10.88 ± 1.01
	GG	70.29 ± 2.19^b^	94.91 ± 3.16	63.79 ± 3.28	94.93 ± 3.32	97.06 ± 3.71	17.94 ± 0.94	62.20 ± 1.83	10.60 ± 1.51
g.51546 T>C (rs319197012)	TT	70.28 ± 2.04	95.15 ± 3.22	63.54 ± 3.19	95.08 ± 3.12	96.88 ± 3.05	17.83 ± 0.95	62.23 ± 1.80^ab^	10.65 ± 1.61
	TC	70.70 ± 1.97	94.65 ± 4.34	64.53 ± 4.54	95.13 ± 3.17	97.05 ± 3.59	17.95 ± 0.93	62.09 ± 2.02^b^	10.89 ± 1.01
	CC	71.00 ± 2.03	95.63 ± 2.97	64.76 ± 3.10	95.88 ± 3.52	97.58 ± 3.35	18.15 ± 0.91	62.91 ± 1.97^a^	10.59 ± 0.93
g.55035 T>C (rs333279910)	TT	70.13 ± 1.99^b^	94.72 ± 2.74	63.66 ± 3.05	94.90 ± 3.08	96.45 ± 3.22	17.78 ± 0.95	62.03 ± 1.65	10.99 ± 0.97^A^
	TC	70.93 ± 1.98^a^	95.04 ± 4.52	64.52 ± 4.39	95.27 ± 3.10	97.41 ± 3.47	18.05 ± 0.94	62.30 ± 2.12	10.75 ± 0.98^AB^
	CC	70.66 ± 2.02^ab^	95.57 ± 3.19	64.40 ± 3.78	95.86 ± 3.70	97.49 ± 3.15	18.00 ± 0.87	62.77 ± 1.97	10.26 ± 2.01^B^
g.55088 C>T (rs322138947)	CC	70.13 ± 1.99^b^	94.72 ± 2.74	63.66 ± 3.05	94.90 ± 3.08	96.45 ± 3.22	17.78 ± 0.95	62.03 ± 1.65	10.99 ± 0.97^A^
	CT	70.93 ± 1.98^a^	95.03 ± 4.52	64.52 ± 4.39	95.27 ± 3.10	97.41 ± 3.47	18.05 ± 0.94	62.30 ± 2.12	10.75 ± 0.98^AB^
	TT	70.6 ± 2.04^ab^	95.57 ± 3.19	64.40 ± 3.78	95.86 ± 3.70	97.49 ± 3.15	18.00 ± 0.87	62.77 ± 1.97	10.26 ± 2.01^B^

Note: BW, Body weight/kg; BL, Body length/cm; BH, Body height/cm; CC, Chest circumference/cm, AC, Abdominal circumference/cm; TC, Tube circumference/cm; LHC, Leg and hip circumference/cm; LBT, Living backfat thickness/mm. The data are expressed as mean ± standard deviation. Different lowercase letters represent significant differences at 0.05 level (*p* < 0.05), different uppercase letters indicate significant differences at 0.01 level (*p* < 0.01)*,* and the same letters (case-insensitive) show no significant difference (*p* > 0.05).

The association between 4 SNP loci g.48810 A>G (rs343060194), g.51546 T>C (rs319197012), g.55035 T>C (rs333279910), and g.55088 C>T (rs322138947)) and 8 growth traits at 12 months (Table 7) was examined using the SPSS 22 software. The results showed that the body length of pigs with the GG genotype at the g.48810 A>G (rs343060194) locus was significantly different from that of the AG genotype (*p* < 0.01). The abdominal circumference of pigs with the AG genotype was significantly different from that of the GG genotype (*p* < 0.05). The body weight and abdominal circumference of pigs with the TC genotype at the g.51546 T>C (rs319197012) intron locus were significantly different from that of the TT genotype (*p* < 0.05), and the same was observed for the leg and hip circumference and living backfat thickness with the TC genotype from that of the TT genotype (*p* < 0.01). The body weight of pigs with the TC genotype at the g.55035 T>C (rs333279910) exon locus was significantly different from that of the TT genotype (*p* < 0.05), and the same was observed for the living backfat thickness with the TC genotype from that of the TT genotype (*p* < 0.01). The body height of pigs with the CC genotype was significantly different from that of the TC and TT genotypes (*p* < 0.05). The leg and hip circumference of pigs with the TC and CC genotypes were significantly different from that of the TT genotype (*p* < 0.05). The body weight of pigs with the CT genotype at the g.55088 C>T (rs322138947) locus was significantly different from that of the CC genotype (*p* < 0.05); and the same was observed for the living backfat thickness with the CT genotype from that of the CC genotype (*p* < 0.01). The body height of pigs with the TT genotype was significantly different from that of the CC and CT genotypes (*p* < 0.05); the leg and hip circumference of pigs with the CT and TT genotypes were significantly different from that of the CC genotype (*p* < 0.05); there were no significant differences in other indicators (*p* > 0.05).

**TABLE 7:** Association analysis between *ACAA1* gene and growth traits of 12-month-old Xiangsu pigs.

SNPS	Genotypes	B W (kg)	B L (cm)	B H (cm)	C C (cm)	A C (cm)	T C (cm)	L H C (cm)	L B T (mm)
g.48810 A>G (rs343060194)	AA	137.72 ± 4.77	130.44 ± 1.39^AB^	72.20 ± 1.29	119.52 ± 1.94	126.88 ± 3.77^ab^	19.64 ± 0.86	87.16 ± 3.08	13.88 ± 1.22
	AG	138.17 ± 4.10	130.04 ± 1.26^B^	71.57 ± 1.48	119.83 ± 1.98	128.14 ± 3.52^a^	19.76 ± 0.79	87.96 ± 2.52	14.18 ± 1.13
	GG	137.12 ± 4.06	130.56 ± 1.17^A^	71.92 ± 1.60	119.91 ± 2.20	126.69 ± 3.43^b^	19.69 ± 1.35	87.19 ± 2.73	14.02 ± 1.04
g.51546 T>C (rs319197012)	TT	136.76 ± 4.38^b^	130.49 ± 1.30	71.72 ± 1.62	119.71 ± 2.11	126.71 ± 3.46^b^	19.60 ± 0.76	86.86 ± 2.88^B^	13.71 ± 1.22^B^
	TC	138.25 ± 3.86^a^	130.20 ± 1.19	71.89 ± 1.51	119.77 ± 1.98	127.87 ± 3.46^a^	19.82 ± 1.40	88.02 ± 2.42^A^	14.37 ± 0.90^A^
	CC	137.88 ± 4.29^ab^	130.39 ± 1.27	71.91 ± 1.35	120.21 ± 2.26	127.03 ± 3.86^ab^	19.67 ± 0.85	87.52 ± 2.80^AB^	14.08 ± 1.04^AB^
g.55035 T>C (rs333279910)	TT	136.66 ± 4.32^b^	130.43 ± 1.26	71.70 ± 1.56^b^	119.94 ± 2.04	126.72 ± 3.60	19.75 ± 1.50	86.78 ± 2.89^b^	13.74 ± 1.19^B^
	TC	138.27 ± 3.97^a^	130.21 ± 1.23	71.68 ± 1.52^b^	119.71 ± 1.98	127.62 ± 3.48	19.73 ± 0.80	87.83 ± 2.50^a^	14.30 ± 0.99^A^
	CC	137.86 ± 4.20^ab^	130.51 ± 1.27	72.40 ± 1.35^a^	119.89 ± 2.39	127.49 ± 3.62	19.57 ± 0.74	88.00 ± 2.66^a^	14.11 ± 1.05^AB^
g.55088 C>T (rs322138947)	CC	136.66 ± 4.32^b^	130.43 ± 1.26	71.70 ± 1.56^b^	119.94 ± 2.04	126.72 ± 3.60	19.75 ± 1.50	86.78 ± 2.89^b^	13.74 ± 1.19^B^
	CT	138.27 ± 3.97^a^	130.21 ± 1.23	71.68 ± 1.52^b^	119.71 ± 1.98	127.62 ± 3.48	19.73 ± 0.80	87.83 ± 2.50^a^	14.30 ± 0.99^A^
	TT	137.86 ± 4.20^ab^	130.51 ± 1.27	72.40 ± 1.35^a^	119.89 ± 2.39	127.49 ± 3.62	19.57 ± 0.74	88.00 ± 2.66^a^	14.11 ± 1.05^AB^

Note: BW, Body weight/kg; BL, Body Length/cm; BH, Body height/cm; CC, Chest circumference/cm; AC, Abdominal circumference/cm; TC, Tube circumference/cm; LHC, Leg and hip circumference/cm; LBT, Living backfat thickness/mm. The data are expressed as mean ± standard deviation. Different lowercase letters represent significant differences at 0.05 level (*p* < 0.05), different uppercase letters indicate significant differences at 0.01 level (*p* < 0.01), and the same letters (case-insensitive) show no significant difference (*p* > 0.05).

### 3.6 Association analysis between *ACAA1* gene diplotypes and growth performance

Association analysis was performed between five diploid combinations and the growth traits of 6-month-old of Xiangsu pigs. Hap1/3, -GCCT/ATTC- outperformed Hap2/2, -GTTC/GTTC- in terms of body weight. Hap1/1, -GCCT/GCCT-was superior to Hap2/2, -GTTC/GTTC- in chest circumference, Hap2/5, -GTTC/GCTC- outperformed Hap2/2, -GTTC/GTTC- in terms of tube circumference, Hap1/1, -GCCT/GCCT-outperformed Hap1/3, -GCCT/ATTC- Hap2/5, -GTTC/GCTC-, Hap2/4, -GTTC/GTCT-in terms of leg and hip circumference (Table 8). The association analysis between five diplotypes and growth traits of 12-month-old Xiangsu pigs revealed that Hap1/1, -GCCT/GCCT-was superior to Hap2/2, -GTTC/GTTC- in body weight and leg and hip circumference. Hap2/4, -GTTC/GTCT-was superior Hap1/3, -GCCT/ATTC- in body length, Hap2/5, -GTTC/GCTC- outperformed Hap2/2, -GTTC/GTTC in terms of body height and tube circumference (Table 9). In a nutshell, Hap1/1, -GCCT/GCCT-can be employed as an advantageous genotype combination for subsequent breeding.

**TABLE 8:** Relationship between diploid types and growth traits at 6 months of age in Xinagsu pigs.

Diplotype	Frequency (%)	B W (kg)	B L (cm)	B H (cm)	C C (cm)	A C (cm)	T C (cm)	L H C (cm)	L B T (mm)
Hap1/3	19.0	71.20 ± 1.69^a^	94.54 ± 5.6	65.34 ± 5.66	95.23 ± 2.49^ab^	97.00 ± 2.73	18.03 ± 0.89^ab^	62.06 ± 2.10^b^	10.82 ± 1.08
Hap2/2	12.0	69.55 ± 2.20^b^	94.22 ± 3.2	63.18 ± 3.29	94.00 ± 2.64^b^	95.77 ± 2.81	17.50 ± 0.91^b^	62.13 ± 1.46^ab^	10.65 ± 1.12
Hap2/5	7.6	70.79 ± 1.85^ab^	95.50 ± 2.9	64.71 ± 2.95	95.93 ± 3.08^ab^	98.21 ± 4.04	18.29 ± 1.07^a^	61.86 ± 1.17^b^	11.27 ± 0.86
Hap2/4	6.0	70.91 ± 2.12^ab^	95.45 ± 3.7	64.45 ± 2.66	95.09 ± 3.14^ab^	97.63 ± 4.06	17.91 ± 1.14^ab^	61.91 ± 1.81^b^	10.59 ± 0.92
Hap1/1	5.4	70.80 ± 2.25^ab^	95.50 ± 3.8	65.60 ± 3.17	96.30 ± 2.50^a^	97.30 ± 3.13	17.90 ± 0.88^ab^	63.40 ± 1.51^a^	10.47 ± 1.07

Note: BW, Body weight/kg; B L, Body length/cm; BH: Body height/cm; CC, Chest circumference/cm; AC, Abdominal circumference/cm; TC, Tube circumference/cm; LHC, Leg and hip circumference/cm; LBT, Living backfat thickness/mm. The data are expressed as mean ± standard deviation.Different lowercase letters represent significant differences at 0.05 level (*p* < 0.05), different uppercase letters indicate significant differences at 0.01 level (*p* < 0.01), and the same letters (case-insensitive) show no significant difference (*p* > 0.05).

**TABLE 9:** Relationship between diploid types and growth traits at 12 months of age in Xinagsu pigs.

Diplotype	Frequency (%)	B W (kg)	B L (cm)	B H (cm)	C C (cm)	A C (cm)	T C (cm)	L H C (cm)	L B T (mm)
Hap1/3	19.0	138.37 ± 3.88^ab^	129.80 ± 1.21^b^	71.60 ± 1.50^ab^	119.77 ± 1.85	128.57 ± 3.08	19.71 ± 0.83^ab^	88.29 ± 2.23^ab^	14.38 ± 0.97
Hap2/2	12.0	135.86 ± 3.91^b^	130.63 ± 1.18^ab^	71.09 ± 1.60^b^	120.45 ± 2.06	126.68 ± 3.26	19.41 ± 0.67^b^	86.41 ± 2.79^b^	13.61 ± 1.08
Hap2/5	7.6	138.00 ± 3.80^ab^	130.43 ± 1.28^ab^	72.50 ± 1.40^a^	120.00 ± 2.04	126.07 ± 3.67	20.50 ± 2.85^a^	87.71 ± 2.55^ab^	14.23 ± 0.98
Hap2/4	6.0	136.91 ± 3.91^ab^	131.18 ± 0.87^a^	72.18 ± 1.66^ab^	119.45 ± 2.25	127.27 ± 3.32	19.73 ± 0.79^ab^	86.73 ± 2.33^ab^	13.90 ± 1.28
Hap1/1	5.4	139.10 ± 3.78^a^	130.60 ± 1.35^ab^	72.30 ± 1.57^ab^	120.40 ± 2.37	128.20 ± 3.58	19.70 ± 0.82^ab^	88.40 ± 2.22^a^	14.40 ± 0.94

Note: BW, Body weight/kg; BL, Body length/cm; BH, Body height/cm; CC, Chest circumference/cm; AC, Abdominal circumference/cm; TC, Tube circumference/cm; LHC, Leg and hip circumference/cm; LBT, Living backfat thickness/mm. The data are expressed as mean ± standard deviation.Different lowercase letters represent significant differences at 0.05 level (*p* < 0.05), different uppercase letters indicate significant differences at 0.01 level (*p* < 0.01), and the same letters (case-insensitive) show no significant difference (*p* > 0.05).

## 4 Discussion

This study investigated and analyzed the expression levels of the *ACAA1* gene in different tissues (heart, liver, spleen, lung, kidney, and longissimus dorsi muscle) of Xiangsu pigs at 6 months of age The results revealed that the *ACAA1* gene was expressed in all examined tissues, including the heart, liver, spleen, lung, kidney, and longissimus dorsi muscle of Xiangsu pigs. Among them, the expression of *ACAA1* gene in liver was significantly different from that in other tissues (*p* < 0.01). ACAA1 regulates fatty acid oxidation and lipid metabolism by catalyzing peroxisomal fatty acid β-oxidation (Luo et al., 2018). Lipid metabolism is tightly linked to fat deposition in muscle, which directly influences the meat taste of pork products. Liver and muscle are the main metabolic organs involved in the regulation of lipid metabolism, and investigation of the expression level and spatial and temporal changes of the *ACAA1* gene in critical visceral organs, and longissimus dorsi muscle of Xiangsu pigs is highly imperative.

We further examined the expression trend of ACAA1 mRNA in longissimus dorsi muscle of Xiangsu pigs at different ages (newborn, 6-month-old and 12-month-old). Interestingly, the expression level of the *ACAA1* gene in the longissimus dorsi muscle decreased with age. Previous studies have shown that gene expression in tissues varies during different growth stages. The intramuscular fat content in the longissimus thoracis muscle of Tibetan sheep shows an increasing trend from 4 months to 1.5 years old (*p* < 0.05), while the *MYH4* gene exhibits differential expression between the longissimus thoracis muscles at 4 months and 1.5 years old (Wen et al., 2022). Transcriptional analysis was conducted on the longissimus dorsi muscle of pigs at different growth stages, identifying many differentially expressed genes (DEGs) related to lipid metabolism and muscle development, the majority of which are involved in intramuscular fat (IMF) deposition (Li et al., 2023). Knockdown of the *ACAA1* gene promoted lipid droplet formation and lipid accumulation in sheep preadipocytes (Wang et al., 2021), inhibiting the *ACAA1* gene expression promoted intramuscular fat deposition in chicken (Li et al., 2019; Xie et al., 2014). In another investigation, upregulated expression of the *ACAA1* gene in mice was revealed to inhibit abdominal fat and liver lipid accumulation in high-fat diet mice (Xie et al., 2014). Therefore, the *ACAA1* gene could influence fat deposition in the longissimus dorsi muscle of Xiangsu pigs at different ages by regulating lipid metabolism.

In the present investigation, we analyzed blood DNA extracted from 184 Xiangsu pigs to determine the effect of *ACAA1* gene polymorphism on fat deposition in the longissimus dorsi muscle of Xiangsu pigs. Amplification of the *ACAA1* gene sequence yielded four SNP loci: g.48810 A>G (rs343060194), g.51546 T>C (rs319197012), g.55035 T>C (rs333279910), and g.55088 C>T (rs322138947). g.51546 T>C (rs319197012) is an intron mutation, g.48810 A>G (rs343060194), g.55035 T>C (rs333279910), and g.55088 C>T (rs322138947) are exon synonymous mutations identified using gene polymorphism parameter evaluation. The four mutation sites were consistent with HWE (*p* > 0.05) and had moderate polymorphism (0.25<*PIC* < 0.50). At the same time, synonymous mutations have been shown to alter mRNA splicing and secondary structure, as well as amino acid co-translation and post-translational folding pathways (Sharma et al., 2019; Supek et al., 2014). Human cancer research has also demonstrated that synonymous mutations potentially change RNA binding proteins and miRNA binding sites (Teng et al., 2020). In this view, it is critical to investigate the association between SNPs in introns and exons of the *ACAA1* gene and backfat deposition in Xiangsu pigs.

LD analysis of the four SNPs revealed that the g.55035 T>C (rs333279910) and g.55088 C>T (rs322138947) had the strongest linkage and belonged to a strong LD (*r* ^2^ = 1.000) (Guryev et al., 2006; Slatkin, 2008). Furthermore, previous studies revealed a strong LD between gene exon mutations, which exert a potential synergistic effect on animal phenotypes (Zhao et al., 2021). Therefore, we hypothesize that the strong linkage mutation sites of g.55035 T>C (rs333279910) and g.55088 C>T (rs322138947) in the *ACAA1* gene may influence pig growth traits.

The relationship between four SNPs of the *ACAA1* gene and the growth traits of Xiangsu pigs revealed that the strong linkage imbalance sites g.55035 T>C (rs333279910) and g.55088 C>T (rs322138947) significantly differed from the body weight (*p* < 0.05) and the living backfat thickness of Xiangsu pigs (*p* < 0.01). Moreover, the heterozygous genotypes of the two loci revealed a dominant genotype in the body weight and living backfat thickness of 12-month-old Xiangsu pigs. These data provided more evidence that these two sites may have a synergistic effect on pig growth and backfat deposition. The g.48810 A>G (rs343060194) locus may primarily influence pig body weight, body length and abdominal circumference, while the g.51546 T>C (rs319197012) locus may influence the growth and backfat deposition of pigs in the later stages of fattening. Emerging evidence indicates that genes related to adipogenesis (Martinez-Montes et al., 2018; Shi et al., 2019) and fatty acid metabolism (Chen et al., 2019; Guo et al., 2017) signaling pathways play a role in pig backfat development (Gozalo-Marcilla et al., 2021) and that pig backfat thickness and body weight are moderately positively correlated (r = 0.632) (Hoa et al., 2021). Diplotypes analysis revealed that Hap1/1, -GCCT/GCCT-were beneficial to growth traits at 6 months of age. Hap1/1, -GCCT/GCCT-, Hap2/5, -GTTC/GCTC- were favorable for growth traits at 12 months of age and could be utilized as advantageous genotype combinations for breeding. The *ACAA1* gene mutations g.55035 T>C (rs333279910) and g.55088 C>T (rs322138947) may be linked to the body weight and living backfat thickness of Xiangsu pigs. Therefore, the *ACAA1* gene is a promising candidate gene for pig growth and development and backfat deposition.

## 5 Conclusion

This study investigated the tissue-specific expression of the *ACAA1* gene in Xiangsu pigs. The results showed that the expression level of *ACAA1* gene mRNA was highest in the liver of 6-month-old pigs. The expression level of *ACAA1* gene mRNA in the longissimus dorsi muscle of Xiangsu pigs decreased with age. In addition, this study conducted an association analysis of *ACAA1* gene SNPs with the growth traits of Xiangsu pigs. The results showed that there are four SNPs in the *ACAA1* gene of Xiangsu pigs; g.55035 T>C (rs333279910) and g.55088 C>T (rs322138947) were strongly linked (*r* ^*2*^ = 1.000). The strong linkage loci exhibited significant differences in body weight and body height and living backfat thickness. These SNPs potentially influence the growth traits of Xiangsu pigs and are valuable SNP markers for improving the growth performance of Xiangsu pigs.

## Data Availability

The original contributions presented in the study are included in the article/Supplementary Materials, further inquiries can be directed to the corresponding author.
