# Supplementary material for: Association analysis between Acetyl-Coenzyme A Acyltransferase-1 gene polymorphism and growth traits in Xiangsu pigs
Source: Front Genet. 2024 May 2;15:1346903. doi: 10.3389/fgene.2024.1346903 (PMC11096523; doi:10.3389/fgene.2024.1346903)
Supplement: Supplementary file 1 [file Table1.docx]

| Tissue | concentration（ng/ul） | A260/A280 | A260/A230 |
| --- | --- | --- | --- |
| Heart（6-month-old） | 1447.2 | 1.86 | 2.07 |
| Liver（6-month-old） | 1508.0 | 1.96 | 2.01 |
| Spleen（6-month-old） | 1547.8 | 1.91 | 2.02 |
| Lung（6-month-old） | 1380.2 | 1.94 | 2.07 |
| Kidney（6-month-old） | 1602.0 | 1.93 | 2.01 |
| Longest dorsal muscle（6-month-old） | 1502.3 | 1.94 | 2.02 |
| Longest dorsal muscle（newborn） | 1527.1 | 1.86 | 2.04 |
| Longest dorsal muscle（12-month-old） | 1104.3 | 1.95 | 2.11 |

Supplementary Table 1 RNA quality detection of heart, liver, spleen, lung, kidney and longissimus dorsi muscle at 6 months old and longissimus dorsi muscle at newborn and 12 months old.
